# Draft Genome Sequence of Desulfovibrio sulfodismutans ThAc01, a Heterotrophic Sulfur-Disproportionating Member of the *Desulfobacterota*

**DOI:** 10.1128/MRA.00202-20

**Published:** 2020-03-26

**Authors:** Lewis M. Ward, Emma Bertran, David T. Johnston

**Affiliations:** a Department of Earth and Planetary Sciences, Harvard University, Cambridge, Massachusetts, USA; b Earth-Life Science Institute, Tokyo Institute of Technology, Tokyo, Japan; c Department of Geosciences, Princeton University, Princeton, New Jersey, USA; University of Southern California

## Abstract

Here, we describe the genome of Desulfovibrio sulfodismutans ThAc01, a *Desulfobacterota* member first isolated from freshwater mud and the first strain reported to be capable of growth via sulfur disproportionation. As such, this genome expands our understanding of the diversity of sulfur-disproportionating microorganisms.

## ANNOUNCEMENT

Desulfovibrio sulfodismutans ThAc01 was first isolated from freshwater marine mud and was the first organism characterized as capable of growth via the disproportionation of either sulfite or thiosulfate to sulfide and sulfate (1, 2). Unlike many sulfur disproportionators that are incapable of growth via sulfate reduction (e.g., see reference 3), D. sulfodismutans is also able to grow via sulfate reduction coupled to the oxidation of small organic compounds, although this does result in slower growth than that during disproportionation (2). D. sulfodismutans was sequenced as part of a larger study to identify genetic markers to distinguish sulfate-reducing organisms from sulfur-disproportionating organisms (4–6).

Purified genomic DNA was ordered from the DSMZ. D. sulfodismutans was grown anaerobically at 35°C in medium 641 prior to DNA extraction at the DSMZ with a JetFlex genomic DNA purification kit from GenoMed. After submission to MicrobesNG, DNA libraries were prepared using a Nextera XT library preparation kit with a Hamilton Microlab STAR automated liquid-handling system. Libraries were sequenced by using an Illumina HiSeq 250-bp paired-end protocol. Adapters were trimmed from reads using Trimmomatic v0.30 (7), and *de novo* assembly was performed using SPAdes v3.7 (8). Annotation was performed using RAST v2.0 (9). Genome completeness was estimated with CheckM v1.0.12 (10), and the likelihood of the presence or absence of metabolic pathways was estimated with MetaPOAP v1.0 (11). The taxonomic assignment of the genome was determined with GTDB-Tk v0.3.2 (12). Hydrogenase proteins were classified with HydDB (13). All software was run using default parameters.

The D. sulfodismutans genome was recovered at 108× coverage as 1,080,467 reads, which were assembled into 295 contigs. The draft genome has an *N*_50_ value of 37,406 bp and totals 4,376,887 bp, with 4,454 coding sequences and 54 RNAs. The genome has a GC content of 63.5%. The genome was determined to be 100% complete and 0.6% redundant and to have 0% strain heterogeneity by CheckM, based on the presence of conserved single-copy marker genes.

Metabolic pathways for sulfur disproportionation are expected to be indistinguishable from those for dissimilatory sulfate reduction (e.g., see reference 14); consistent with this expectation, the D. sulfodismutans genome encodes a full dissimilatory sulfate reduction pathway, including sulfate adenylyltransferase, adenylylsulfate reductase, dissimilatory sulfite reductase, and the sulfite reduction-associated DsrMKJOP complex. The D. sulfodismutans genome encodes a group A FeFe hydrogenase and a group 4e NiFe hydrogenase, as determined by HydDB. The D. sulfodismutans genome encodes a flagellum, consistent with the description of D. sulfodismutans as a motile organism (2). While truncation of the C-terminal domain of AprB was recently proposed as a marker for sulfur disproportionation in diverse bacteria (4), this marker is not present in D. sulfodismutans (i.e., the genome encodes a full-length AprB). This trait may be related to the ability of D. sulfodismutans to grow facultatively as a sulfur disproportionator or a sulfate reducer, in contrast to obligate sulfur-disproportionating organisms in the genus *Desulfobulbus*, the genomes of which encode the truncated AprB.

Taxonomic assignment by GTDB-Tk places D. sulfodismutans in the *Desulfovibrionaceae* family of the *Desulfobacterota* phylum (formerly Deltaproteobacteria); however, GTDB-Tk does not place D. sulfodismutans within the genus *Desulfovibrio* but instead suggests that it may represent a separate novel genus-level lineage and therefore may require taxonomic reassignment.

### Data availability.

This whole-genome shotgun project has been deposited at DDBJ/ENA/GenBank under accession number JAAGRQ00000000. The FASTQ files of the raw reads were deposited in the NCBI SRA under accession number SRR11035950.
